# Oligodendroglioma of the Hippocampus: A Case Report and Systematic Review on Therapeutic Approaches of Oligodendroglioma After WHO 2021 Classification

**DOI:** 10.3390/ph18030349

**Published:** 2025-02-28

**Authors:** Panagiotis Skouras, Georgios Giakoumettis, Charalampos Argyros, George Vavoulis, Emmanouil K. Verigos, Dimitrios Giakoumettis

**Affiliations:** 1 Department of Biological Chemistry, Medical School, National and Kapodistrian University of Athens, 11527 Athens, Greece; panosskouras@hotmail.gr; 2 Department of Social and Family Medicine, General Hospital of Karditsa, 43100 Thessaly, Greece; 3 Medical Physics & Digital Innovation Laboratory, School of Medicine, Faculty of Health Sciences, AHEPA University Hospital, Aristotle University of Thessaloniki, 54636 Thessaloniki, Greece; 4 Department of Neurosurgery, “Agios Savvas” General Anticancer-Oncological Hospital of Athens, 11522 Athens, Greece; xargyros@gmail.com; 5 Department of Neurosurgery, “KAT” General Hospital of Athens, 14561 Kifissia, Greece; georgevavoulismd@gmail.com; 6 Department of Radiotherapy, “Agios Savvas” General Anticancer-Oncological Hospital of Athens, 11522 Athens, Greece; manolisverigos@gmail.com; 7 Department of Neurosurgery, Democritus University of Thrace, 68100 Alexandroupolis, Greece

**Keywords:** glioma, oligodendroglioma, molecular diagnostics, IDH-mutated, 1p/19q co-deletion

## Abstract

**Background:** Oligodendrogliomas are a molecularly distinct subtype of glioma according to the WHO 2021 tumor classification, defined as isocitrate dehydrogenase (IDH) mutations and 1p/19q co-deletion. This updated classification has changed the approach to glioma management by emphasizing the critical role of molecular diagnostics. This study explores current therapeutic strategies for adult oligodendrogliomas and contextualizes findings with a patient with a Grade 3 oligodendroglioma of the hippocampus. **Methods:** A systematic review was conducted, synthesizing evidence from 36 studies published between 2021 and 2024. The review focuses on surgical resection, PCV chemotherapy (procarbazine, lomustine, vincristine), and radiotherapy, with progression-free survival (PFS) and overall survival (OS) as primary outcomes. Moreover, a 45-year-old woman diagnosed with an IDH-mutant, 1p/19q-co-deleted Grade 3 oligodendroglioma is presented to illustrate clinical management. **Results:** The review highlights the significance of molecular profiling in personalizing treatment strategies. The findings highlight that maximal safe surgical resection combined with PCV chemotherapy and radiotherapy optimizes PFS and OS. However, our case underwent chemotherapy and radiotherapy after a multidisciplinary consultation, demonstrating favorable initial outcomes. These findings reaffirm the importance of integrating molecular insight into clinical decision-making. **Conclusions:** Advancements in molecular diagnostics have profoundly enhanced the personalization of therapy for oligodendrogliomas, yielding improved survival outcomes. Optimal management should entail a multidisciplinary approach incorporating surgery, chemotherapy, and radiotherapy, guided by molecular features. This study reinforces the necessity of molecular-driven strategies to improve survival and quality of care for patients with oligodendroglioma.

## 1. Introduction

Gliomas, originating from neuroglial cells in the brain and spinal cord, account for over 80% of malignant central nervous system (CNS) tumors. The 2021 World Health Organization (WHO) classification incorporates advancements in molecular biology, categorizing gliomas into adult-type diffuse gliomas, pediatric-type diffuse low-grade and high-grade gliomas, circumscribed astrocytic gliomas, and ependymal tumors [1,2,3]. In the updated classification, oligodendrogliomas are defined by the presence of isocitrate dehydrogenase IDH1 or IDH2 mutations and 1p/19q co-deletion. Despite this molecular specificity, predicting outcomes remains challenging, as survival data from older histology-based studies and registries are confounded by the inclusion of 20–70% of patients lacking these molecular markers. Based on the latest report of the Central Brain Tumor Registry of the United States (CBTRUS) 2016–2020, the average annual age-adjusted incidence rate of IDH-mutant and 1p/19q-co-deleted oligodendroglioma is 0.29 (95% CI 0.28–0.30). The 5-year survival rate for all ages for patients with oligodendroglioma and anaplastic oligodendroglioma is 84.6% (95% CI 83.7–85.4) and 66.8 (95% CI 65.2–68.4), respectively [4]. Improved survival has been linked to extensive yet safe surgical resection and the use of procarbazine, CCNU (lomustine), and vincristine (PCV) chemotherapy in combination with partial brain radiotherapy [5]. The current study focuses on optimizing treatment strategies, including therapies targeting IDH mutations and refining cytotoxic regimens. In addition, we review current therapeutic approaches in adults based on the WHO 2021 classification of gliomas. Moreover, we report the case of a 45-year-old woman with an oligodendroglioma located in the left hippocampus.

## 2. Materials and Methods

### 2.1. Case Presentation

**Figure 1:**
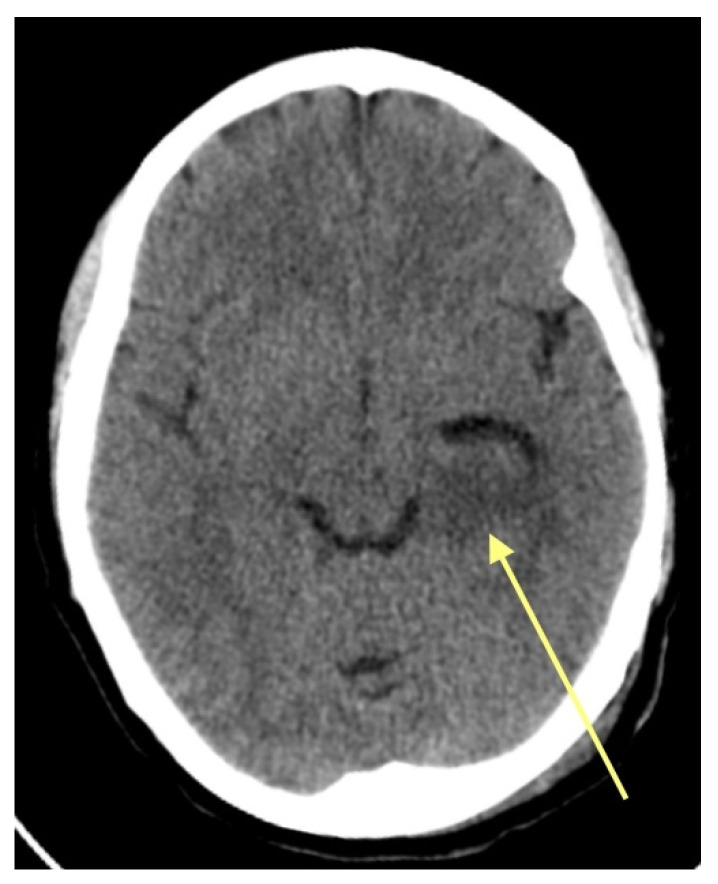
Axial CT image. The yellow arrow shows the hypodense area at the area of the left hippocampus.

A 45-year-old female patient presented in the ED due to dysarthria, drooping of the mouth corner, and an epileptic episode that had occurred 4 h ago. She was a right-handed teacher with no significant medical history. She was evaluated by a neurologist who initially considered the possibility of an ischemic stroke in the differential diagnosis. A CT scan of the brain (Figure 1) was performed, which revealed a hypodense area in the left hippocampus. Subsequently, the patient was referred to the neurosurgical team. During the examination, the patient showed improvement in her dysarthria and did not present any other neurological deficits. There were no memory impairments and no focal neurological deficits. The patient was admitted for further investigation with an MRI of the brain. Signed informed consent was received from the patient and, since this is a retrospective reflection of the case, an Institutional Review Board Statement was not deemed necessary.

### 2.2. Systematic Review

The search strategy included three databases, Pubmed, Scopus and ClinicalTrials, where an advanced search was undertaken including the keywords “oligodendrogliomas” AND “therapy” for the period from June 2021 to July 2024. This period was chosen due to the publication of *The 2021 WHO Classification of Tumors of the Central Nervous System: A Summary* on 29 June 2021. The 2021 WHO grading, introduced in the fifth edition published in August 2021, brought significant changes, advancing the role of molecular diagnostics in CNS tumor classification, particularly for oligodendrogliomas. As this is a systematic review of the literature, an Institutional Review Board Statement was not deemed necessary. PRISMA guidelines and the PICO framework were followed.

### 2.3. Review Structure and PICO Framework

The review is structured using the following PICO framework:

- Population (P): Adults (≥18 years) diagnosed with oligodendroglioma, as defined by the WHO 2021 classification (IDH-mutant, 1p/19q-co-deleted, Grade 2 or 3).
- Intervention (I): Treatments including surgery, chemotherapy, radiotherapy, or other therapeutic interventions.
- Comparison (C): No specific comparator.
- Outcome (O): Progression-free survival (PFS) and/or overall survival (OS).

This update will include studies that meet predefined inclusion and exclusion criteria.

### 2.4. Inclusion Criteria

Eligible studies must focus on adult patients (≥18 years) diagnosed with oligodendroglioma, as defined by the 2021 World Health Organization (WHO) classification, specifically IDH-mutant and 1p/19q-co-deleted Grade 2 or 3 gliomas. We will include original studies such as randomized clinical trials (RCTs), non-RCTs, observational studies, retrospective studies, meta-analyses, and case series involving more than three patients. Studies must be published in English, evaluate treatment modalities (e.g., surgery, chemotherapy, radiotherapy, or other therapies), and report outcomes related to progression-free survival (PFS) or overall survival (OS). Only studies published within the last three years, after the adoption of the WHO 2021 classification, will be considered.

### 2.5. Exclusion Criteria

We will exclude studies that do not meet the inclusion criteria, including reviews, systematic reviews, case reports, letters to the editor, animal studies, and gray literature. Studies focused on pediatric patients (<18 years), written in languages other than English, or addressing diagnoses other than oligodendroglioma based on the 2021 WHO molecular classification will also be excluded. Furthermore, studies that do not address treatment approaches for oligodendrogliomas will not be considered.

### 2.6. Data Extraction

Following the search and removal of duplicates, the research team will independently screen the titles and abstracts of the identified studies to determine their potential inclusion. The full texts of all potentially relevant studies will then be assessed against our inclusion criteria. Any disagreements between reviewers regarding a study’s eligibility will be resolved by consulting a sixth reviewer. Data will be extracted from the included studies using a standardized, piloted form. The extracted data will include study design, population details, the number of participants, comprehensive descriptions of the analytical techniques used, and study results.

## 3. Results

### 3.1. Case Results

**Figure 2:**
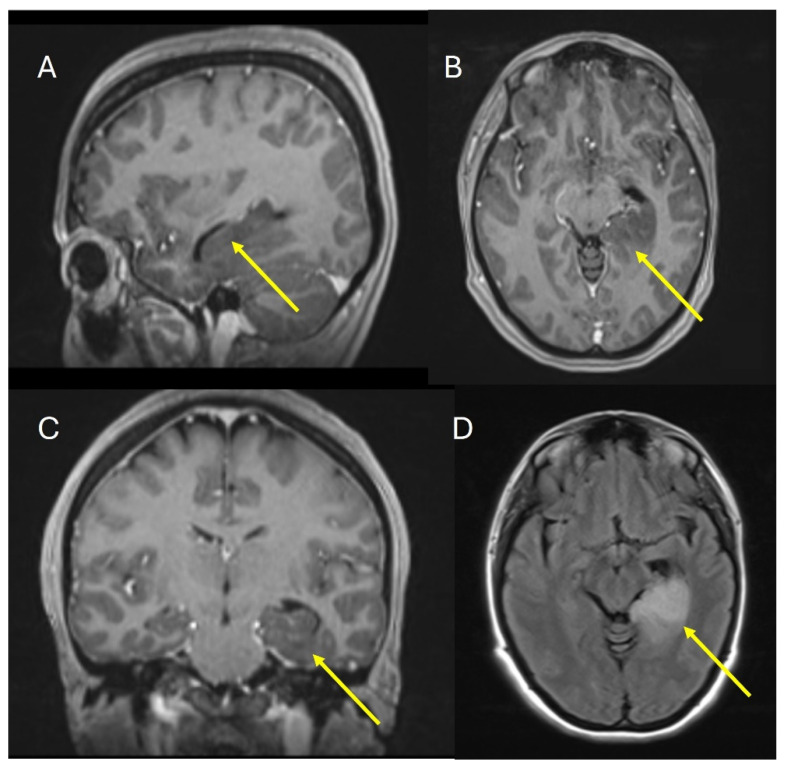
MRI of the brain. (**A**) Saggital image T1WI, (**B**) Axial image T1WI, (**C**) Coronal image T1WI, (**D**) FLAIR axial image. The yellow arrow shows the hippocampal formation and parahippocampal gyrus.

The patient underwent an MRI of the brain with contrast, which revealed a lesion occupying the left hippocampal formation and the parahippocampal gyrus. The lesion was not contrast enhanced; it was hypo-intensive in T1WI and high-intensive in FLAIR and T2WI (Figure 2). The patient was offered and underwent a biopsy of the lesion.

The pathology report revealed an IDH-mutated (IDH1^R132H^) and 1p/19q co-deleted oligodendroglioma, Grade 3 (WHO grading 2021). The tumor was characterized by a moderate-to-sufficient number of cells with relative morphological uniformity. There was no evidence of vascular hyperplasia or necrosis. No H3.3 K27M mutation was found. Additionally, the neoplastic cells expressed the transcription factors Olig2, FOXG1, and GFAP. There was no expression of the mutated p53 protein, and the nuclear expression of ATRX was preserved. There was no expression of the FUBP1 protein and no overexpression of the EGFR protein. After the diagnosis and treatment options were discussed with the patient and her family, she decided to continue with oncological therapy, including chemotherapy and radiotherapy, receiving the typical Stupp protocol. At her 3-month follow-up, there were no clinical signs or symptoms from her tumor, and she had only mild chemotherapy-related side effects. This aligns with the review’s findings, indicating a favorable prognosis for IDH-mutant, 1p/19q co-deleted oligodendrogliomas when treated with multimodal therapy.

### 3.2. Systematic Review Results

**Figure 3:**
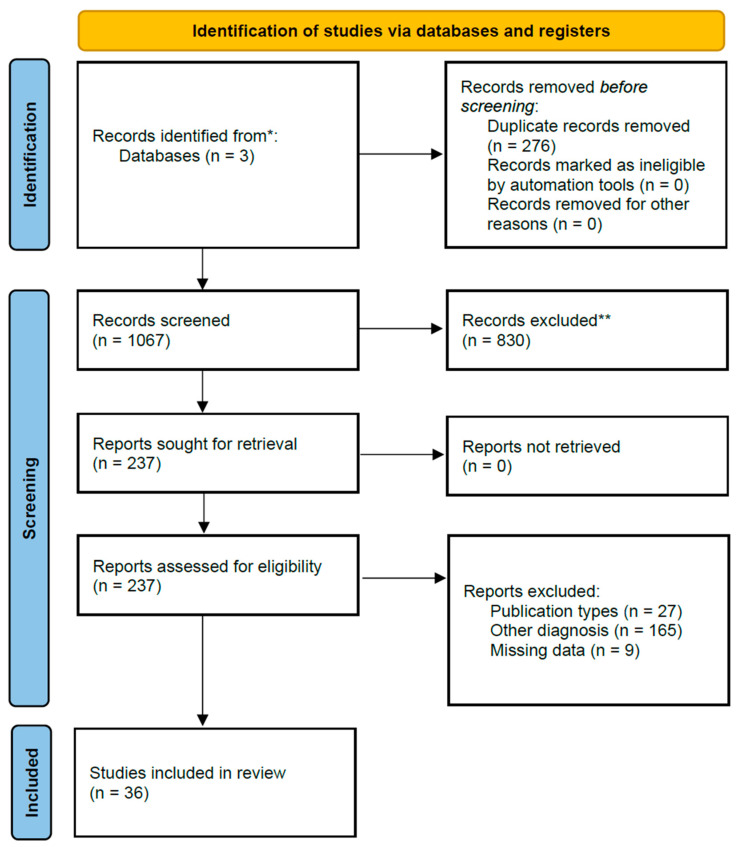
PRISMA 2020 flow diagram for new systematic reviews which included searches of databases and registers only [6]. * Pubmed: 409, Scopus: 927, Clinical Trials: 7. ** No automation tools were used.

**Figure 4:**
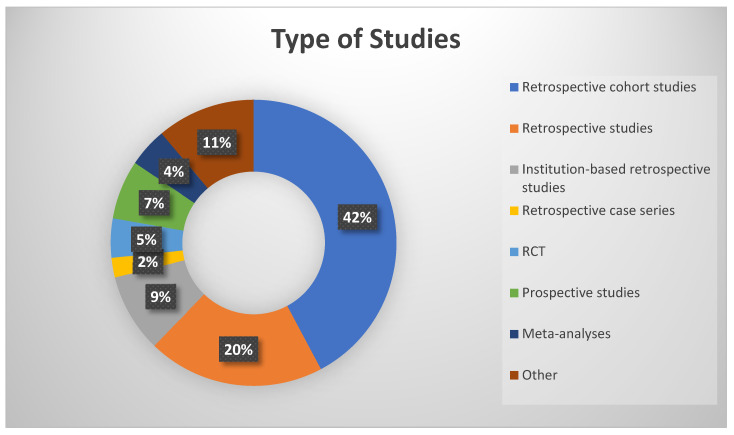
Types of study included.

We conducted a comprehensive search using three databases. The search in PubMed identified 409 studies, and in Scopus, 927 studies were found, all from the period between 2021 and 2024. Additionally, a focused search was performed on ClinicalTrials.gov, restricted to completed trials, and only studies with available results were considered within the timeframe for study completion 1 January 2021 to 20 July 2024. This approach ensured that only the most relevant and up-to-date clinical trials, focusing on treatment strategies for oligodendroglioma in adult populations, were included. The search in ClinicalTrials.gov yielded seven studies. The total number of articles identified across all databases was 1343. These articles were analyzed using Rayyan accessed on 20 July 2024 (https://new.rayyan.ai/), and 276 duplicates were removed. The remaining 1067 articles were screened based on their titles and abstracts. After applying the inclusion and exclusion criteria, 830 articles were excluded. The remaining 237 articles underwent full-text screening and, ultimately, 36 studies were included in the final analysis (Appendix A, Table A1) (Figure 3, PRISMA flow chart). The types of study included are shown in Figure 4. The total number of patients was 7134 with an IDH mutated and 1p/19q co-deleted oligodendroglioma. The majority of patients were between 35 and 65 years old, with a mean age of approximately 45.7 years and range from 19 to 83 years. The pooled mean overall survival (OS) and progression-free survival (PFS) were not possible to extract or extrapolate safely due to the fact that there was no uniform report on them. Several studies within the cohort highlighted that median OS values exceeded 100 months for certain patient subgroups and others reported on 1-, 3- and 5-year overall survival. Survival outcomes, particularly PFS and OS, were found to vary significantly based on molecular characteristics, such as IDH mutation status and 1p/19q co-deletion. However, a trend seems to exist on age, which, above 35 years, was associated with decreased OS in some studies, and treatment factors, including the extent of surgery, chemotherapy, and radiotherapy, which played a crucial role in influencing survival outcomes.

## 4. Discussion

Oligodendroglioma of the hippocampus is rare, and the literature is scarce in this region. Our patient was diagnosed with an IDH-mutant, 1p/19q-co-deleted Grade 3 oligodendroglioma, according to the WHO 2021 classification. This molecular signature is a key diagnostic and prognostic marker, commonly observed in reviewed cases. The tumor was located in the hippocampus, an uncommon site for oligodendrogliomas, which can complicate surgical resection and impact treatment strategies. However, the survival is still based on the molecular characteristics of the tumor, in addition to the extent of resection (EOR), and oncological therapies. This systematic review provides an analysis for oligodendrogliomas defined by the WHO 2021 CNS tumor classification. All of the 36 studies included in the analysis highlighted the important role of the molecular markers, IDH mutations and 1p/19q co-deletion, in predicting therapeutic response and survival outcomes. The therapeutic strategies presented included surgical resection with adjuvant chemotherapy, and radiotherapy as the cornerstone of treatment. Nevertheless, chemotherapy and radiotherapy without surgical excision may also be a therapeutic choice, with temozolomide and PCV regimens as standard chemotherapy options. In our case, a biopsy was performed, followed by chemotherapy and radiotherapy. While PCV (procarbazine, lomustine, vincristine) is a standard regimen, the patient received temozolomide (TMZ), a well-tolerated alternative commonly used in recent studies. Moreover, adjuvant therapies, particularly the combination of radiotherapy and chemotherapy, have also demonstrated significant survival benefits, with better outcomes in high-grade tumors. Even though all studies agree on the importance of molecular markers, there are significant variations in treatment protocols as well as patient outcomes. This stresses the need for a standardized approach and report. There is no doubt that molecular markers play and will continue to play a pivotal role in the diagnosis, prognosis, and treatment of oligodendrogliomas [7,8,9], as depicted in all studies. In addition, elevated FXYD2 mRNA expression and MGMT (O6-Methylguanine-DNA Methyltransferase) promoter methylation were highlighted in several studies as improving survival outcomes [10,11]. In general, median OS has been shown to be better for patients with IDH-mutant and 1p/19q co-deleted tumors, exceeding 14 years in some cohorts [12]. Nevertheless, several factors such as age, tumor size, incomplete resection, or certain genetic mutations have been blamed for negatively influencing survival [5,13], e.g., a mutation on PIK3CA along with intratumoral calcifications was linked to worse prognosis [14,15]. The emerging use of DNA methylation profiling and gene signatures demonstrated their potential to stratify patients and predict treatment responses [8,16]. In terms of surgical operation, the extent of resection and, more specifically, gross total resection or subtotal resection, has been strongly associated with overall survival and progression-free survival [8,9,12,17]. In a retrospective study by Hervey-Jumper et al., 190 patients (48.5%) had an IDH-mutant and 1p/19q-co-deleted oligodendroglioma. The median age of this group was 42.6 years, with a range between 34.4 and 49.5 years. Tumor locations included the frontal lobe in 116 patients (61%), the temporal lobe in 21 patients (11.1%), the parietal lobe in 25 patients (13.2%), the insular lobe in 27 patients (14.2%), and other locations in 1 patient (0.5%). In terms of treatment, all patients received a surgical operation, 88 patients (47.1%) received chemotherapy (out of which, 76 patients received TMZ), and 72 patients (37.9%) received radiotherapy. Achieving an EOR of at least 75% for OS and 80% for PFS is particularly beneficial. Outcomes showed a median progression-free survival (PFS) of 11.69 years, with a range between 9.29 and 17.70 years [7]. Chemotherapy and radiotherapy are another therapeutic choice for patients. The combination of them, particularly using PCV (procarbazine, lomustine, vincristine) or temozolomide (TMZ), has shown better results in PFS and OS in comparison to monotherapy, as shown in the clinical trial CODEL and the randomized controlled trial by Bush et al. [18,19]. The choice between these modalities may, however, depend on tumor grade, e.g., Grade 2 tumors with favorable features could be tackled with chemotherapy alone to reduce long-term toxicities [19,20]. Adjuvant radiotherapy has been shown in the meta-analysis by Koh to improve OS and PFS in Grade 3 tumors, whereas proton radiotherapy, though effective, has been associated with several risks such as radiation-induced contrast enhancements (RICE), particularly in lower-grade tumors [21]. Radiotherapy alone has not been proven to improve OS and PFS in comparison to its combination with other treatment modalities [18]. Finally, emerging therapies have offered hope for better therapies. Mutant IDH inhibitors induce differentiation into astrocytic-like states, while NOTCH1 mutations may serve as biomarkers for response stratification. Recent studies suggest that mutant IDH inhibitors promote differentiation of oligodendroglioma cells into astrocytic-like states, reducing tumor proliferation [22]. Unlike conventional chemotherapy, which induces direct cytotoxicity, IDH inhibitors work by modifying tumor metabolism. Early clinical trials have shown disease stabilization in IDH-mutant gliomas, but long-term survival benefits remain uncertain, as most trials are still in Phase I/II. Meanwhile, other studies have offered insight into the use of advanced imaging techniques, such as FET-PET (Fluoroethyltyrosine Positron Emission Tomography), for assessing treatment response and guiding clinical decisions [23]. Targeted therapy has also been shown to be a promising future option. The retrospective study by Jun et al. on recurrent high-grade gliomas treated with anlotinib included 29 patients with a median age of 50 years, ranging from 15 to 71 years. Of these, 93.1% had undergone surgery prior to the study. Tumor characteristics revealed that 48.3% of cases were multifocal or disseminated, while 51.7% were focal. The molecular characteristics of the patients included in the study indicated that 79.3% were IDH wild type, while only 20.7% had an IDH mutation. A methylated MGMT promoter status was reported in 41.4% and unmethylated in 58.6% of patients. Anlotinib is a multi-targeted tyrosine kinase inhibitor (TKI) that was administered as monotherapy or in combination with other therapies, including temozolomide, semustine, irinotecan, and vemurafenib. All patients had received radiotherapy with concurrent chemotherapy during their initial treatment after diagnosis of high-grade glioma, and previous therapies included bevacizumab and nimotuzumab in some patients. The outcomes showed a median progression-free survival (PFS) of 9.4 months (95% CI: 6.5–12.3), with a 6-month PFS rate of 62.1%. Median overall survival (OS) was 12.7 months (95% CI: 9.7–15.7), with a 6-month OS rate of 79.3% and a 1-year OS rate of 48.3%. The key findings of this study suggest that anlotinib, a multi-target anti-angiogenic agent, demonstrated potential efficacy as both monotherapy and in combination therapy for the treatment of recurrent high-grade gliomas [24]. Moreover, other studies investigate the use of neoadjuvant and adjuvant immunotherapy in patients with a second or third recurrence of histologically confirmed IDH1R132H-positive, 1p/19q-co-deleted oligodendrogliomas (CNS WHO Grade 2 or 3). This immunotherapy approach aims to harness IDH1 mutation-specific vaccination combined with checkpoint inhibition to improve patient outcomes. Checkpoint inhibitors targeting PD-1/PD-L1 and IDH1-specific vaccines have shown potential in preclinical models of IDH-mutant gliomas (Table 1) [25].

However, unlike in other cancers, immunotherapy has shown mixed results in gliomas due to their highly immunosuppressive microenvironment. Combining checkpoint inhibitors with radiotherapy or chemotherapy may enhance response rates, but more clinical data is needed before replacing conventional therapies. Two large meta-analyses with many patients have also been included in our study. Xuan et al. present a systematic review and meta-analysis that examines the outcomes of treatments in IDH-mutated and 1p/19q co-deleted oligodendrogliomas. The review included data from 17 studies with a total of 11,949 participants, out of which 1646 patients had IDH-mutated and 1p/19q co-deleted oligodendrogliomas. The tumor samples were mostly obtained after gross total resection (GTR) and subtotal resection (STR), and from biopsies in some cases. The treatments and interventions analyzed in the review primarily involved chemotherapy, with PCV (procarbazine, lomustine, vincristine) or temozolomide (TMZ) being the most commonly used agents. The latter was favored in more recent studies due to its better tolerability. Standard protocols were followed for both regimens. Where RT was combined with chemotherapy either concurrently or sequentially, better outcomes were seen compared to monotherapy. Additional RT or chemotherapy were used for patients who experienced disease progression, as salvage therapy. The outcomes of the review showed that adjuvant RT improved PFS by 50% (hazard ratio [HR] 0.52; confidence interval [CI]: 0.40–0.66), where both Grade 2 and Grade 3 oligodendroglioma patients benefited from this improvement. Adjuvant RT also improved OS by 28% (HR 0.72; CI: 0.56–0.93), with a more pronounced benefit in Grade 3 oligodendroglioma patients compared to Grade 2 [26]. The second meta-analysis by Zhang et al., involved 1944 patients with newly diagnosed gliomas, including 169 patients with oligodendrogliomas. The median age range of the cohort was between 54.2 and 59 years, although some studies included younger patients, with age ranges starting as low as 20.5 years. The study did not provide specific details on the extent of surgical resection or tumor locations. Chemoradiotherapy included temozolomide (TMZ) and conventional External Beam Radiotherapy (EBRT) following the established Stupp protocol [27]. Hypofractionation was used in some cases. The study also highlighted the frequent use of glucocorticoids to manage edema, though doses exceeding 2 mg/day were associated with worsened lymphopenia. Outcomes showed that severe lymphopenia significantly worsened overall survival (OS), with a hazard ratio of 1.99 (95% CI: 1.74–2.27). OS was also influenced by radiation dose and chemotherapy regimens. However, molecular characteristics such as 1p/19q co-deletion and MGMT promoter methylation were not consistently reported across studies. This meta-analysis underscored the importance of lymphopenia in predicting OS outcomes in glioma patients, though more molecular data would be valuable for a clearer understanding of prognostic factors [28].

## 5. Limitations

This review is subject to certain limitations. First, the predominance of retrospective studies limits the ability to draw definitive causal inferences. Second, the heterogeneity in treatment protocols and patient populations across studies complicates direct comparisons. In addition, there is no uniform way of presenting the outcomes; where several studies report either median or year OS and PFS, others do not. Moreover, only a few studies included long-term follow-up, particularly in emerging therapies, leaving uncertainties about their sustained efficacy and safety. Even though it was not in the scope of this systematic review, a pooled analysis was not possible to carry out safely due to the diversity of the reported outcomes.

## 6. Conclusions

Advancements in molecular diagnostics have profoundly shaped the management of oligodendrogliomas, reinforcing the necessity of a personalized, multidisciplinary approach. Maximal safe surgical resection should be the primary goal, as it significantly improves progression-free survival (PFS) and overall survival (OS). In cases where complete resection is not achievable, subtotal resection followed by adjuvant therapy remains a viable option. Patients with IDH-mutant, 1p/19q-co-deleted oligodendrogliomas should be considered for adjuvant chemotherapy and/or radiotherapy based on tumor grade and patient-specific factors. Given the prognostic and predictive significance of IDH mutations and 1p/19q co-deletion, molecular profiling should be integrated into routine clinical practice. Additional markers, such as MGMT promoter methylation and PIK3CA mutations, may further refine treatment strategies. Regular MRI monitoring is essential for the early detection of recurrence or progression. Personalized follow-up intervals should be based on initial treatment response and molecular risk stratification. IDH inhibitors and immunotherapy hold promise as adjuncts or alternatives in refractory or recurrent cases. Clinicians should consider enrolling eligible patients in clinical trials evaluating these novel therapies to expand treatment options. By integrating molecular insights into clinical decision-making, adopting a patient-centered approach, and considering novel therapies when appropriate, clinicians can optimize outcomes and quality of life for patients with oligodendrogliomas. Future prospective or retrospective studies about oligodendroglioma patients focusing on standardizing treatment protocols and reporting results in a uniform way—including percentage of resection, overall survival rate, and progression-free survival rate—would be the next logical thing to do.

## Figures and Tables

**Table 1:** Comparison of IDH inhibitors, immunotherapy and conventional treatments.

Treatment Approach	Mechanism of Action	Clinical Benefits	Limitations
Surgery + Radiotherapy + Chemotherapy (PCV/TMZ)	Surgical resection reduces tumor burden; radiation and chemotherapy target remaining tumor cells	Proven survival benefits; standard of care for decades	Long-term toxicities (e.g., cognitive decline, radiation necrosis)
IDH Inhibitors (Mutant IDH1/2 Inhibitors)	Block mutant IDH enzyme, reducing production of oncometabolite D-2-hydroxyglutarate (D-2HG)	Induce tumor differentiation; prolong disease stability in early-phase trials	Limited Phase III trial data; unclear long-term efficacy
Immunotherapy (Checkpoint Inhibitors + IDH1 Vaccine)	Boosts immune response against glioma cells by targeting PD-1/PD-L1 pathways or training immune cells against IDH mutations	Promising results in preclinical and early-phase clinical trials	Mixed efficacy in gliomas; risk of immune-related adverse events

## Data Availability

Data are contained within the article.

## Abbreviations

The following abbreviations are used in this manuscript:

ALA	Aminolevulinic Acid
CBTRUS	Central Brain Tumor Registry of the United States
CI	Confidence Interval
CNS	Central Nervous System
CT	Computed Tomography
EBRT	External Beam Radiotherapy
ED	Emergency Department
EOR	Extent of Resection
FET-PET	Fluoroethyltyrosine Positron Emission Tomography
FLAIR	Fluid-Attenuated Inversion Recovery
GTR	Gross Total Resection
HR	Hazard Ratio
IDH	Isocitrate Dehydrogenase
MGMT	O6-Methylguanine-DNA Methyltransferase
MRI	Magnetic Resonance Imaging
OS	Overall Survival
PCV	Procarbazine, CCNU (lomustine), and Vincristine
PFS	Progression-Free Survival
PICO	Population, Intervention, Comparison, Outcome
RCTs	Randomized Clinical Trials
RICE	Radiation-Induced Contrast Enhancements
STR	Subtotal Resection
T1WI	T1-Weighted Imaging
TKI	Tyrosine Kinase Inhibitor
TMZ	Temozolomide
WHO	World Health Organization

## Appendix A

**Table A1:** Studies included in the systematic review.

PMID	Authors	Year of Publication	(N) IDH Mutant and 1p/19q Co-Deletion	Treatment Protocol	Overall Survival	Progression-Free Survival	Key Points
32886916	Pierina Navarria, Federico Pessina et al. [9]	2021	42	The study treated patients with temozolomide (TMZ) and radiotherapy (RT). TMZ alone was given to 12 patients, while 18 received RT with concomitant TMZ followed by adjuvant TMZ. RT (60 Gy in 2 Gy fractions) was given to 12 patients with adjuvant TMZ. TMZ dosing was 75 mg/m^2^ daily during RT and 150–200 mg/m^2^ for five days every 28 days in the adjuvant phase, up to 12 cycles or until disease progression.	1 yr OS = 97.6, 3 yr OS = 88.8%, 5 yr OS = 85.4	Median PFS = 76 months (32–89) PFS for 1 yr =92.9%–3 yr = 63.4%–5 yr = 63.4%	In addition to IDH status, EOR and RTV significantly impact survival. Adjuvant radiotherapy is crucial for all AG patients. The study highlights the need to consider multiple factors in treatment planning for AG patients.
35998208	Maximilian J. Mair, Annette Leibetseder, et al. [8]	2022	201	In the early postoperative treatment group (83 of 201 patients), 56 received radiotherapy (RT) with chemotherapy, 12 had chemotherapy only, and 15 had RT alone. Temozolomide was given to 64 of 68 patients, while 2 received RT with PCV (procarbazine, lomustine, vincristine). One patient had bevacizumab (Grade 3), and six received other/experimental treatments.	1. unadj.median OS =182 mo (129 not reached) for patients who received early postop. treatment 2. median OS = 217 mo (188 not reached) for the group who did not receive it	1. PFS = 96.1 months with early postop treatment and 2. PFS = 69.0 months for those who did not receive it	The study found that early postoperative treatment did not improve progression-free survival (PFS) or overall survival (OS) in patients with CNS WHO Grade 2 and 3 oligodendroglioma. DNA methylation profiling revealed potential predictive value for treatment response, suggesting the need for further validation in prospective trials.
36599113	Shawn L Hervey-Jumper, Yalan Zhang, et al. [7]	2023	190	Out of 88 patients (47.1%), 76 (40.6%) received temozolomide, and 72 (37.9%) underwent radiotherapy. No data were available for other oncological therapies.	Median OS yrs NA (22.2 to NA)	Median PFS yrs 11.69 (9.29 to 17.70)	The study found that a greater extent of surgical tumor resection (EOR) in patients with diffuse low-grade glioma (LGG) significantly improves overall survival (OS) and progression-free survival (PFS), with EOR beginning at 75% improving OS and at 80% improving PFS. The findings emphasize the importance of maximal resection while preserving neurological function, particularly benefiting patients with astrocytoma and oligodendroglioma.
32678879	Kurt A. Jaeckle, Karla V. Ballman, et al. [18]	2021	36	Temozolomide (TMZ) was given at 75 mg/m^2^/day during radiotherapy and 150–200 mg/m^2^ on days 1–5 every 28 days for up to 12 cycles. Radiotherapy was conventionally fractionated at a total dose of 5940 cGy in 33 fractions.	Not statistically different between the arms due to limited sample size and follow-up	Arm A + B (RT-based): Not reached at median follow-up of 6.6 years. Arm C (TMZ alone): Median PFS = 2.9 years	The initial CODEL phase III trial found that patients with newly diagnosed 1p/19q co-deleted anaplastic oligodendroglial tumors treated with TMZ alone experienced significantly shorter progression-free survival (PFS) compared to those treated with radiotherapy (RT) or RT plus TMZ. Consequently, the ongoing CODEL trial has been redesigned to compare RT plus PCV versus RT plus TMZ.
38579724	Avishay Spitzer, Simon Gritsch, et al. [22]	2024	3	IDH inhibitors (ivosidenib and vorasidenib) were used for treatment.	Not explicitly mentioned but implied long-term stability due to PFS durations.	Patient MGH170: Stable disease 60 months post-treatment. Patient MGH229: Stable disease 55 months post-treatment. Patient BWH445: Stable disease 66 months post-treatment.	Mutant IDH inhibitors induce lineage differentiation in IDH-mutant oligodendroglioma, favoring astrocytic-like states over stem/progenitor-like states, and depleting stem-like cells. NOTCH1 mutations can modify this response, potentially serving as a biomarker for patient stratification.
38981364	Zhi Xuan Ng, Eng Siew Koh, et al. [26]	2024	1646	Treatment involved PCV or temozolomide (TMZ), with TMZ preferred for better tolerability. Adjuvant radiotherapy was given at 59.4 Gy. Combination therapy (RT and chemotherapy) showed better outcomes, and salvage RT or chemotherapy was used for progression.	Adjuvant RT improved OS by 28% overall (HR 0.72, CI: 0.56–0.93). OS benefits were more pronounced in Grade 3 than in Grade 2 oligodendroglioma.	Adjuvant RT improved PFS by 50% (HR 0.52, CI: 0.40–0.66). Both Grade 2 and Grade 3 oligodendroglioma patients benefited in terms of PFS.	Adjuvant radiotherapy (RT) improves overall survival (OS) and progression-free survival (PFS) in patients with oligodendroglioma, particularly in those with Grade 3 tumors, compared to no adjuvant RT. However, for patients with low-risk features such as Grade 2 tumors and gross total resection, alternative approaches like adjuvant chemotherapy alone may be reasonable due to the lack of significant survival benefit from adjuvant RT.
37216045	Luisa Allwohn, Josy Wolfgang, et al. [29]	2023	114	Temozolomide (TMZ) was the most common treatment, with PC and PCV as alternatives. Median cycles were 12 for TMZ, 6 for PC, and 4.5 for PCV. Conformal radiotherapy was given at a median dose of 59.4 Gy (range 54–67.5 Gy). A watch-and-wait approach was used for low-risk cases, while salvage therapies, including re-surgery or re-RT with systemic treatments, were applied for progression.	Median OS: 236.0 months. OS rates at 2, 4, and 6 years: 99.0%, 97.9%, and 96.2%, respectively.	Median PFS: 66.9 months. PFS rates at 2, 4, and 6 years: 89.5%, 76.3%, and 46.0%, respectively.	This study analyzed the impact of various treatments on progression-free survival (PFS) and overall survival (OS) in a homogeneous cohort of patients with 1p/19q-co-deleted and IDH-mutant oligodendrogliomas, finding that radiochemotherapy (RCT) significantly prolonged PFS in WHO Grade 3 tumors. The results suggest that surgical resection, radiotherapy, and chemotherapy are beneficial for WHO Grade 2 tumors, while RCT, often using temozolomide, is particularly effective for WHO Grade 3 tumors.
37877960	Alexandra M Giantini-Larsen, Zaki Abou-Mrad, et al. [30]	2024	21	Temozolomide (TMZ) was the primary chemotherapy, typically started 1.9 months post-surgery following standard dosing protocols. External beam radiation therapy (EBRT) was initiated at a median of 2.1 months post-surgery, with doses generally ranging from 54 to 60 Gy. Postoperatively, 84% of patients received antiepileptic drugs (mainly levetiracetam), and steroids were used with a low complication rate (8% hyperglycemia).	2-year OS: 83.1%. 5-year OS: 69.7%. Median OS for astrocytoma IDHmt: 6.38 years. Median OS for oligodendroglioma IDHmt: Not reached	Not directly reported, but overall trends show significant survival benefits with GTR/NTR and appropriate adjuvant therapy	The study found that elderly patients (≥65 years) with IDH1-mutant gliomas have favorable survival rates compared to their IDH-wild-type counterparts, with 2- and 5-year survival rates of 83.1% and 69.7%, respectively. Despite high rates of medical comorbidities, most patients underwent craniotomy for resection, and the majority received postoperative chemotherapy and radiation therapy, highlighting the importance of not using age alone to determine prognosis or treatment decisions.
35948444	Lauren R. Schaff, Marina Kushnirsky, et al. [31]	2022	20	Treatment included olaparib (150 mg, three times per week) and temozolomide (TMZ) at 50–75 mg/m^2^ daily, given continuously or in 6-week cycles with 2–3 weeks off. Most patients had prior radiotherapy, with one receiving it concurrently, though specific doses were not detailed. Prior therapies included bevacizumab, IDH inhibitors (ivosidenib and vorasidenib), PCV, CCNU, and experimental agents.	Not provided	Grade 2–3 IDH-mutant gliomas: Median PFS = 7.8 months. Grade 4 IDH-mutant gliomas: Median PFS = 1.3 months. IDH-wild-type gliomas: Median PFS = 2.0 months	In patients with IDH-mutant oligodendroglioma treated with the combination of olaparib and temozolomide (TMZ), partial responses were observed, with progression-free survival (PFS) ranging from 1.6 to 8.7 months. Common side effects included fatigue, gastrointestinal symptoms, and hematologic toxicity, with some patients requiring dose adjustments or discontinuation due to toxicity.
37611077	Connor J. Kinslow, Ali I. Rae, et al. [11]	2023	1297, MGMT-methylated: 1009, MGMT-unmethylated: 288 Group 1: Chemotherapy: 938 (72.3%) received chemotherapy Group 2: Radiation: 670 (66%) received radiotherapy	Single-agent regimens, mainly temozolomide (TMZ), were given to 83.5% of patients, while multiagent regimens, mostly PCV, were given to 14.3%. TMZ was administered at 150–200 mg/m^2^ daily for 5 days in a 28-day cycle, and PCV followed specific dosing for each agent. Conventional external beam radiotherapy (EBRT) was typically given at 54–60 Gy, but specific fractionation schedules were not detailed. Most chemotherapy patients also received radiotherapy, and future de-escalation trials may refine treatment approaches.	5-year OS rates: MGMT-methylated tumors: 81%. MGMT unmethylated tumors: 70%. Chemotherapy + MGMT methylation: Adjusted hazard ratio for survival: 2.61 (indicating better outcomes with methylation)	Not provided	Oligodendrogliomas with 1p/19q-co-deletion and MGMT promoter methylation (mMGMT) show improved overall survival (OS) when treated with chemotherapy, compared to those with unmethylated MGMT (uMGMT). This suggests that MGMT promoter status should be considered in future clinical trials and treatment decisions for these tumors.
37968476	Qinghui Zhu, Haihui Jiang, et al. [15]	2024	305	Chemotherapy regimens were more commonly given to patients with calcified oligodendrogliomas (ODGs). Conventional radiotherapy was used, typically with doses ranging from 54 to 60 Gy in fractions, though specifics were not provided. Calcified tumors were more likely to receive aggressive chemoradiotherapy, highlighting the need for tailored treatment approaches.	Patients with T2-hypointense calcifications had a lower OS compared to those with non-hypointense calcifications. Calcifications associated with cysts were found to be linked to more aggressive tumor characteristics and the worst outcomes.	ODGs with calcification had shorter PFS compared to non-calcified tumors. Calcification was identified as a negative prognostic factor for PFS.	Intratumoral calcification in oligodendrogliomas (ODGs) is not only a diagnostic marker but also a negative prognostic factor for progression-free survival (PFS) and overall survival (OS), with calcified ODGs showing larger tumor diameters, higher tumor grades, and lower resection rates. Additionally, ODGs with T2 hypointense calcifications or concurrent cysts are associated with worse prognoses, indicating the importance of these features in guiding personalized treatment and predicting patient outcomes.
34125374	Nancy Ann Oberheim Bush, Jacob S. Young, et al. [19]	2021	40	Temozolomide (TMZ) was the main chemotherapy used. Patients who received TMZ alone had an average of 13.2 cycles (range: 6–40), while those treated with both TMZ and radiation had an average of 9 cycles (range: 1–18). Standard-dose radiation was typically given concurrently with TMZ, though exact dosages were not specified. At progression, treatments included radiation, additional chemotherapy, or surgical interventions.	Median OS was not reached, indicating extended survival rates. Median PFS for combined radiation and TMZ: 157.8 months. Median PFS for TMZ alone: 31.5 months.	56.3 months	Anaplastic oligodendrogliomas are high-grade gliomas characterized by 1p19q co-deletion, typically treated with surgical resection followed by radiation and chemotherapy. This study found that while upfront radiation and chemotherapy significantly prolong progression-free survival compared to chemotherapy alone, there is no significant difference in overall survival, suggesting that initial chemotherapy alone may be a viable option to avoid the long-term toxicities of radiation.
37603235	Louise Carstam, Francesco Latini, et al. [32]	2023	126	Temozolomide (TMZ) was the most commonly used treatment, accounting for 29.4% of first-line treatments, followed by the PCV regimen (19.0%) and CCNU (lomustine) at 14.3%. Radiotherapy was part of the treatment for 71.4% of patients. A combination of radiotherapy and chemotherapy (either PCV or TMZ) was frequently used, and some patients underwent re-operations during follow-up.	17.8 years	Not provided	Oligodendrogliomas, defined by IDH mutation and 1p19q-co-deletion, are slow-growing CNS tumors with a median survival of nearly 18 years, often presenting with seizures in young to middle-aged adults. Key factors associated with shorter survival include advanced age, larger tumor size, and poor preoperative functional status, while surgical strategy does not significantly impact survival.
36273731	Kepeng Liu, Xiaozu Liao, et al. [20]	2023	1826	Chemotherapy (CT) drugs were not specified, and the specifics of chemoradiation therapy (CRT) regimens were not detailed. Radiation therapy was included as part of CRT, but the types used were not specified. No additional therapies beyond CT and CRT were mentioned.	Median OS for CT group: 146 months. Median OS for CRT group: 111 months	Not provided	Oligodendroglioma is a type of glioma classified by the WHO as Grade 2 or 3, originating from oligodendrocytes in the brain’s white matter, and is known for its invasive nature and variable prognosis. This study found that adjuvant chemotherapy alone after resection provides better overall survival benefits compared to chemoradiation therapy, especially in patients who are younger or older, male or female, white, with tumors in the frontal or parietal lobes, smaller tumor size, and those who underwent gross total resection.
37370678	Julia Gilhodes, Adèle Meola, et al. [16]	2023	68	PCV (procarbazine, lomustine, vincristine) and temozolomide (TMZ) were used in treatment. Concomitant or adjuvant RT-TMZ and RT-PCV were administered. Gene expression analysis identified 8 genes linked to shorter progression-free survival, suggesting potential therapeutic targets to improve outcomes.	OS rates: 12 months: 98.5%, 24 months: 89.7%, 36 months: 88.2%, 48 months: 86.7%	Median PFS: 60.3 months (95% CI: 41.0–not reached). PFS rates: 12 months: 88.2%. 24 months: 70.6%. 36 months: 63.2%. 48 months: 60.1%	Oligodendrogliomas with IDH mutations and 1p/19q co-deletions are associated with a better prognosis but show heterogeneous responses to treatment. A study identified an eight-gene signature linked to poor progression-free survival, suggesting that these genes could help identify patients needing more intensive treatment.
35179134	Antonio Dono, Kristin Alfaro-Munoz, et al. [14]	2022	107	Temozolomide (TMZ) was used in 68% of patients, while other regimens like lomustine/procarbazine/vincristine were used in 6%. Chemotherapy cycles were distributed as follows: ≥12 cycles in 44%, 6–11 cycles in 13%, 1–5 cycles in 12%, and 31% received no chemotherapy. Adjuvant radiotherapy (RT) was administered to 76% of patients. The combination of RT and TMZ did not show better survival benefits than RT alone. PIK3CA mutations were linked to poorer survival outcomes and may help predict response to targeted therapies involving PI3K/AKT/mTOR inhibitors.	Median OS: 165.4 months (13.8 years). Patients with PIK3CA mutations had significantly shorter OS (128.5 months) compared to wild-type (180.8 months)	Median PFS: 79.5 months. PFS was worse for WHO Grade 3 tumors compared to Grade 2 tumors (71 months vs. 83 months)	Oligodendrogliomas, characterized by IDH1/IDH2 mutations and 1p/19q-co-deletion, show worse overall survival when harboring PIK3CA mutations, with no significant improvement in survival from adjuvant temozolomide or radiation therapy. The study suggests that PIK3CA mutations are associated with poorer outcomes, highlighting the need for further evaluation of targeted therapies in these cases.
37435019	Jun Yin, Wenya Yin, et al. [24]	2023	3	Anlotinib was given as monotherapy or in combination with various treatments: temozolomide (TMZ) at 100 mg/m^2^ orally for 7 days every 2 weeks, semustine at 0.1 g/m^2^ every 6–8 weeks, irinotecan at 125 mg/m^2^ intravenously every 2 weeks, and vemurafenib at 960 mg orally every 12 h. All patients had received radiotherapy with concurrent chemotherapy after their high-grade glioma diagnosis. Anlotinib, a multi-target anti-angiogenic agent, showed potential as both monotherapy and combination therapy. Some patients had had previous treatments with bevacizumab and nimotuzumab.	Median OS: 12.7 months (95% CI: 9.7–15.7). 6-month OS rate: 79.3%. 1-year OS rate: 48.3%	Median PFS: 9.4 months (95% CI: 6.5–12.3). 6-month PFS rate: 62.1%	In the study, three patients with anaplastic oligodendroglioma were included, and they were part of the overall cohort of 29 patients treated with anlotinib-containing regimens. The specific outcomes for these patients were not separately detailed, but the overall results showed a median progression-free survival (PFS) of 9.4 months and a median overall survival (OS) of 12.7 months for the entire cohort.
38971940	Yukyeng Byeon, Chaejin Lee, et al. [33]	2024	138	Procarbazine, lomustine, and vincristine (PCV) was used in 1.4% of cases, as was temozolomide (TMZ), while ICE (ifosfamide, carboplatin, and etoposide) was used in 1.4%. Radiotherapy (RT) was used as an adjuvant treatment in 21.7% of cases. Postoperative management included observation for 72.5% of patients, adjuvant therapy (RT or chemotherapy) for 27.5%, and combined radiochemotherapy (RCTx) for 4.3%.	Median OS: 18.4 years, 5-Year OS Rate: 95.5%, 10-Year OS Rate: 76.1%	Median PFS: 6.8 years, 5-Year PFS Rate: 60.1%, 10-Year PFS Rate: 27.4%	Oligodendrogliomas (ODGs) are a rare subtype of diffuse lower-grade gliomas characterized by slow growth and a favorable prognosis, with a median overall survival of 18.4 years. Key prognostic factors include the extent of resection, with gross total resection or subtotal resection with less than 10% residual tumor significantly improving progression-free and overall survival, and the presence of contrast enhancement on MRI, which is associated with worse outcomes.
34513462	Masashi Mizumoto, Hsiang-Kuang Liang, et al. [34]	2021	43	Concurrent chemotherapy included daily temozolomide (TMZ) at 75 mg/m^2^ for 28 days and nimustine (ACNU) at 80 mg/m^2^, administered in two cycles during radiotherapy. Postoperative radiotherapy was conducted using three-dimensional conformal radiotherapy (3D-CRT), with a total dose of 60 Gy in 30 fractions. The planning target volume (PTV) included the surgical cavity and surrounding edema with a 1.2 cm margin.	5-year OS: Anaplastic oligodendroglioma (AO): 76.8%. Anaplastic astrocytoma (AA): 46.1%. Total or partial resection: 72.7%. Biopsy Only: 21.0%. Mean OS: 95.3 months	5-year PFS: Total or partial resection: 52.9%. Biopsy Only: 10.7%. Mean PFS: 66.8 months	Anaplastic oligodendroglioma (AO) patients had a significantly better prognosis compared to anaplastic astrocytoma (AA) patients, with a five-year overall survival (OS) rate of 76.8% versus 46.1%. Additionally, AO cases showed a higher three-year progression-free survival (PFS) rate of 62.8% compared to 41.1% for AA cases, indicating a more favorable outcome for AO.
36598613	Tanja Eichkorn, Jonathan W. Lischalk, et al. [21]	2023	81	Temozolomide (TMZ) was used in 56.2% of cases, PCV (procarbazine, lomustine, vincristine) in 25.8%, and 12.4% received no chemotherapy. Patients received Proton Radiotherapy (PRT), which offers better dose distribution and reduced toxicity compared to photon radiotherapy. For WHO Grade 2 gliomas, the median dose was 54 Gy (range: 50.4–60 Gy), while for WHO Grade 3 gliomas, it was 60 Gy (range: 54–60 Gy), delivered in 1.8–2 Gy fractions. RICE (Radiation-Induced Contrast Enhancement) occurred in 25% of patients, treated with corticosteroids or bevacizumab for symptoms. Re-irradiation and re-chemotherapy were used in 27% and 88% of recurrence cases, respectively.	5-year OS: WHO Grade 2: 85%. WHO Grade 3: 67%	5-year PFS: WHO Grade 2: 60%. WHO Grade 3: 30%	Oligodendroglioma, a subtype of IDH-mutated glioma, was present in 39% of WHO Grade 2 and 45% of WHO Grade 3 cases in the study. The study found that proton radiotherapy (PRT) was effective for treating these tumors, with a 5-year overall survival rate of 85% for WHO Grade 2 and 67% for WHO Grade 3, though the risk of radiation-induced contrast enhancements (RICE) was higher in WHO Grade 2 tumors.
37215951	Saksham Gupta, Noah L. Nawabi, et al. [12]	2023	80	Postoperative chemotherapy was administered as follows: temozolomide (TMZ) in 88.9% of cases and PCV (procarbazine, lomustine, and vincristine) in 4.9%. Adjuvant radiotherapy was provided to 68.8% of patients after surgery. In cases of progression or recurrence, repeat surgery improved survival outcomes, and chemotherapy and radiotherapy were used in 72.1% and 44.2% of cases, respectively.	Median OS: 14.1 years (95% CI for OS was provided)	Median PFS: 5.6 years (for patients experiencing progression)	Grade 3 1p/19q co-deleted oligodendrogliomas are rare primary CNS tumors that often progress or recur after initial treatment, with a median overall survival of 14.1 years. Repeat surgery after progression is associated with increased survival, but not with time to subsequent progression, and decreased survival is linked to low preoperative Karnofsky Performance Status, lack of gross total resection, and persistent postoperative neurologic deficits.
38334907	Obada T. Alhalabi, Philip Dao Trong, et al. [35]	2024	9	The majority of patients received temozolomide (TMZ) at 91%, with additional agents like lomustine (CCNU), etoposide (VP-16), bevacizumab, and tumor peptide vaccines used in smaller percentages. Most patients underwent radiotherapy prior to repeat surgery: 83% received one course, 14% received two courses, and 3% received three courses. Targeted therapies, including mTOR inhibitors, CDK 4/6 inhibitors, PARP inhibitors, and MEK inhibitors, were administered based on molecular diagnostics, with 30% of patients starting targeted therapy.	Median OS after repeat surgery for the cohort: 399 days	Median survival data provided instead of PFS: Across all tumors: 399 days. Glioblastoma IDH-wild-type: 348 days after repeat surgery	Oligodendroglioma, IDH-mutant, was diagnosed in 9 patients (13%) in the study, with WHO Grades 2 and 3. These tumors were primarily located in the frontal and temporal lobes, and patients had undergone various treatments including surgery, radiotherapy, and chemotherapy prior to repeat surgery for molecularly informed targeted therapy.
36215231	Michael M. Wollring, Jan-Michael Werner, et al. [23]	2022	6	Lomustine or lomustine combined with PC was used, with a 42-day cycle for lomustine alone or a 56-day cycle for lomustine+PC.	23.9 months	KPS > 70% all, oligo 18 months	Oligodendroglioma, particularly WHO CNS Grade 3 with IDH mutation and 1p/19q co-deletion, is one of the glioma subtypes evaluated in the study for response to lomustine-based chemotherapy. The study found that FET PET metrics and RANO criteria provide complementary information for assessing treatment response, which can aid in clinical decision-making for continuing or discontinuing chemotherapy.
38845694	Colin M. Harari, Adam R. Burr, et al. [36]	2024	16	Concurrent with PRDR 10, followed by PRDR 10. The PRDR dose was 54 Gy (range 2–60) with a delivery rate of 0.067 Gy/min, in contrast to the usual RT dose of 4–6 Gy.	OS since PRDR: 27 mo (~10%).	PFS since PRDR 6.2 mo. Stratified by recurrent grade: 2 22 mo 50%/22%); 3 12.6 mo (23%/9%); 4 7.3 mo (6%/0%) KPS 100 (16); 90 (22); 80 (12); 70 (2); 60 (2); 50 (4).	Oligodendrogliomas, which accounted for 40% of the patient cohort, were treated with pulsed reduced-dose rate (PRDR) re-irradiation, showing a median overall survival of 12.6 months post-PRDR. The presence of 1p/19q co-deletion, found in 28% of patients, was associated with improved survival outcomes.
36852494	Andrew J. Scott, Luis O. Correa, et al. [37]	2023	20	A total of 77.8% of patients received TMZ sometime after resection, while 75% received radiotherapy (RT) sometime after resection.	10.8 y/~90%	Not provided	Oligodendrogliomas with IDH mutations (IDHmut) are metabolically distinct from IDH wild-type glioblastomas (IDHwt GBMs) and generally have a more favorable prognosis, with a median survival time of approximately 11 years. These tumors are characterized by specific metabolic profiles that can inform patient outcomes and potentially guide targeted therapies.
37147958	Dilek Erdem, Meral Gunaldi, et al. [38]	2023	7	Chemoradiotherapy was used in 76.5% (n = 26) of cases, radiotherapy (RT) in 5.9% (n = 2), and no treatment was applied in 17.6% (n = 6) of cases. RT was used in 5.9% (n = 2) of cases.	A total of 55.9% (n = 19) of the patients were alive at the end of the follow-up period and 44.1% (n = 15) had expired by that time. Duration of follow-up ranged from 15 to 49 months, with an average of 26.75 ± 17.16 months.	Not provided	Oligodendroglioma, a type of brain tumor, was found in 20.6% of the patients in the study, with DDR1 expression levels in both tissue and serum samples being higher compared to the control group, although not statistically significant. The study suggests that DDR1 could be a potential biomarker for brain tumors, including oligodendroglioma, due to its correlation with tumor size and survival rates.
34952000	Pierina Navarria, Federico Pessina, et al. [39]	2021	11	TMZ was administered starting on the first day of HSRT and continued throughout the entire treatment. In the adjuvant setting, rescue treatments included TMZ, lomustine, or bevacizumab. TMZ was given at 75 mg/m^2^ from the first day of HSRT until the completion of the treatment. For radiation, patients received either single-dose stereotactic radiosurgery (SRS) with 25 Gy/1 fraction or hypofractionated stereotactic radiation therapy (HSRT), which included 37.5 Gy in 5 fractions (7.5 Gy per fraction) or 49.5 Gy in 15 fractions (3.3 Gy per fraction). The treatment was tailored for lesions greater than 2 cm, those in the eloquent area, or near critical structures, with HSRT dosed at 49.5 Gy/15 fractions.	Median OS 33 (18–56) months, 1 y 84.0 (±7.3) months, 2 y 59.3 (±9.9) months, 3 y 47.9 (±10.8) months (*p* value 0.0021)	Median PFS 20 (13–49) months, 1 y PFS 72.0 (±8.9) months, 2 y PFS 47.1 (±10.1) months, 3 y PFS 42.4 (±10.2) months (*p* value 0.0087)	Oligodendroglioma patients in the study had a median overall survival of 33 months, with 1, 2, and 3-year survival rates of 84%, 59.3%, and 47.9%, respectively. These patients, particularly those with 1p19q co-deleted IDH mutant WHO Grade 3 gliomas, showed a better prognosis compared to other high-grade glioma types.
35692047	A. Wick, A. Sander, et al. [5]	2022	364	PCV chemotherapy cycles are approximately 6 weeks long. Day 1 involves CCNU (110 mg/m^2^ orally, capped at 200 mg), Days 8 and 29 involve vincristine (1.4 mg/m^2^ intravenously, capped at 2 mg), and Days 8 to 21 involve procarbazine (60 mg/m^2^ orally, capped at 100 mg). For CETEG, CCNU/temozolomide chemotherapy cycles also last 6 weeks. Day 1 includes CCNU (100 mg/m^2^ orally, capped at 200 mg), and Days 2–6 involve temozolomide (100 mg/m^2^ in cycle 1, with dose escalation in 50 mg/m^2^ steps based on toxicity in subsequent cycles). For WHO Grade 2 oligodendrogliomas, radiotherapy is given in 28 fractions of 1.8 Gy, totaling 50.4/54 Gy over 5–6 weeks. For WHO Grade 3 oligodendrogliomas, radiotherapy is delivered in 33 fractions of 1.8 Gy, totaling 59.4 Gy, with one fraction given daily, five days a week, for 6 to 7 weeks.	10 years	Not provided	Oligodendrogliomas are brain tumors characterized by IDH1 or IDH2 mutations and 1p/19q co-deletion, with treatment strategies aiming to balance efficacy and minimize long-term cognitive and functional impairments. The NOA-18 trial seeks to improve qualified overall survival by comparing standard chemoradiation with PCV to an initial chemotherapy regimen with lomustine and temozolomide, delaying radiotherapy to reduce side effects.
35171292	Gaia Ninatti, Martina Sollini, et al. [40]	2022	93	A total of 25 patients (29%) received chemotherapy, 8 patients (9%) received radiotherapy, and 10 patients (11%) received both chemotherapy and radiotherapy.	Not provided	53.5 months	Preoperative [11C]MET PET uptake was more common in oligodendrogliomas than IDH-mutant astrocytomas, with 87% of oligodendrogliomas showing positive uptake. Grade 3 oligodendrogliomas had the highest median TBRmax (3.22), and the extent of resection was a significant independent predictor of progression-free survival in these patients.
36691100	Hongbo Liu, Lu Zhang, et al. [1]	2023	10	Concurrent chemotherapy with TMZ should be administered daily at a dose of 75 mg/m^2^ during radiotherapy (RT). After RT, adjuvant chemotherapy with TMZ (150–200 mg/m^2^ every 28 days) was scheduled for a minimum of 6 cycles, continuing if the patient’s physical condition allowed. If the patient’s condition deteriorated or the disease advanced, the dose was reduced, or chemotherapy was suspended. Post-operative RT was administered within 42 days of surgery, with a total dose of 60 Gy delivered in 2 Gy fractions, Monday to Friday, over 6 weeks.	Median OS was NR (Not Reached)	33.5 months	Oligodendroglioma patients in the study all experienced local recurrences, with no significant difference in progression-free survival (PFS) and overall survival (OS) between the two radiotherapy plans. This suggests that the inclusion of peritumoral edema in the target volume does not impact the recurrence pattern or prognosis for oligodendroglioma.
35058869	Zhang Y, Chen S et al. [28]	2021	1944 patients, 169 oligodendroglioma	The study focusses on the standard Stupp protocol. Alternative regimens are dose-dense TMZ, hypofractionated RT and TMZ, proton therapy and TMZ, PCV, bevacizumab, and dexamethasone.	Overall survival is expressed as Hazard Ratio of 2	Not provided	Lymphopenia nearly doubles the risk of mortality in glioma patients (HR~2.0). The findings are consistent across multiple studies, showing lymphopenia is a negative prognostic factor on OS.
38259678	Mashal Shah, Saad Bin Anis, et al. [41]	2024	32	Chemotherapy (either temozolomide or procarbazine, lomustine, and vincristine [PCV]) was provided to 141 patients. Radiation therapy was provided to 265 patients, and all those who underwent radiation therapy also received chemotherapy (265 patients).	AOs have the longest mean OS (39.8 months), whereas glioblastomas have the shortest mean OS (14.1 months). Median overall survival, 39.8 months for pts with AO.	Not provided	Anaplastic oligodendroglioma (AO) patients had the longest mean overall survival (OS) of 39.8 months among high-grade gliomas in the study, with better outcomes associated with 1p19q co-deletion and IDH mutations. The study found no significant difference in survival between patients treated with PCV (procarbazine, lomustine, and vincristine) and those treated with temozolomide.
35716310	Jan-Michael Werner, Lena Wolf, et al. [42]	2022	2	Regorafenib was administered at 160 mg once daily for the first 3 weeks of each four-week cycle, with individual dose adjustments based on side effects. Of the two patients with oligodendroglioma (OLG) and favorable outcomes (overall survival >12 months) despite early regorafenib discontinuation, one patient received 6 cycles of temozolomide chemotherapy (150–200 mg/m^2^ on days 1–5 of a 28-day cycle) after regorafenib.	Of the two patients with OLG and favorable outcome (OS > 12 months) despite early regorafenib discontinuation, one patient received 6 cycles of temozolomide chemotherapy (150–200 mg/m2 on day 1–5 of a 28-day cycle) after regorafenib.	Not provided	In the study, two patients with oligodendroglioma (IDH-mutant, 1p/19q-co-deleted, WHO CNS Grade 3) showed favorable outcomes, with overall survival exceeding 12 months despite early discontinuation of regorafenib. One patient continued with temozolomide chemotherapy after regorafenib, while the other had no further treatment and showed no progression over one year.
37625215	Arshad A. Pandith, Wani Zahoor, et al. [13]	2023	21	Once the pathology was confirmed, all the patients were started on temozolomide according to the hospital protocol. They were also put on radiotherapy as per the protocol, with concurrent chemo-radiotherapy including temozolomide and megavoltage radiation therapy.	Mean OS 62.4 months for OLG	Mean PFS 40.1 months for OLG	Oligodendroglioma patients with 1p/19q co-deletion exhibit a better prognosis, enhanced response to therapy, and longer overall survival and progression-free survival compared to those without the deletion. This co-deletion is a common early genetic event in oligodendroglial tumors and is now considered a standard of care marker for therapeutic decisions.
36964732	Sidonie Sauvageot, Julien Boetto, et al. [43]	2023	40	A total of 102 patients underwent chemotherapy, with a median interval of 14 months (IQR 2–26 months) between surgery and the first chemotherapy. Temozolomide was administered to 85 patients; procarbazine, CCNU, and vincristine (PCV) to 16 patients; and bevacizumab to 1 patient. Fifty-five percent of patients underwent radiotherapy, with a median interval of 38.5 months (IQR 18–59.8 months) between surgery and radiotherapy.	OS rates at 1, 5, and 10 years were 100% (95% CI 0.99–1), 80% (95% CI 0.72–0. 9), and 58% (95% CI 0.45–0.73), respectively. The median OS was 138 months (Figure 3 and Figure 4). All deaths were disease related. There was a significance tendency for decreased OS associated with preoperative tumor volume > 150 cm^3^, age > 35 years, and EOR < 90%. At last follow-up, 69.7% of patients were still alive, with a median follow-up of 62 months (IQR 36–99 months).	Not provided	Oligodendrogliomas, which can be either WHO Grade 2 or Grade 3, were found in 45 patients in the study, with 26 being Grade 2 and 19 having foci of Grade 3. These tumors showed favorable long-term outcomes with a significant survival benefit when the residual tumor volume was less than 15 cm^3^, and most patients underwent adjuvant chemotherapy and/or radiotherapy post-surgery.
34753441	Kaijia Zhou, Tao Jiang, et al. [10]	2021	114	A total of 392 patients underwent chemotherapy, while 124 did not. Temozolomide (TMZ) was used. A total of 408 patients received radiotherapy, and 108 did not.	Expression of FXYD2 mRNA was higher in patients with a good prognosis, including those with primary glioma (*p* = 0.00031), oligodendroglioma (*p* = 5.6 × 10^−10^), lower WHO grade (*p* = 0.00011), IDH mutation (*p* = 2.5 × 10^−18^), 1p/19q co-deletion (*p* = 5.3 × 10^−12^), and Oligodendroglioma, IDH-mutant and 1p/19q-co-deleted (*p* = 2.3 × 10^−20^).	Subgroup analysis showed that different subgroups of glioma patients with high FXYD2 mRNA expression also had longer OS. Among them, low-grade glioma (*p* = 0.011), high-grade glioma (*p* = 0.000), oligodendroglioma (*p* = 0.004)	Oligodendroglioma, characterized by IDH mutation and 1p/19q co-deletion, shows higher FXYD2 mRNA expression compared to other glioma types, indicating a better prognosis. Patients with oligodendroglioma and high FXYD2 mRNA expression have longer overall survival and are more likely to respond positively to temozolomide chemotherapy.

**Table A2:** Study Stratification Based on Oxford CEBM Level of Evidence.

PMID	Authors	Study Type	Level of Evidence
32678879	Kurt A. Jaeckle, Karla V. Ballman, et al. [18]	Randomized Controlled Trial (RCT)	Level 1
32886916	Pierina Navarria, Federico Pessina et al. [9]	Retrospective Observational	Level 3
34125374	Nancy Ann Oberheim Bush, Jacob S. Young, et al. [19]	Retrospective Cohort Study	Level 3
34513462	Masashi Mizumoto, Hsiang-Kuang Liang, et al. [34]	Retrospective Observational	Level 3
34753441	Kaijia Zhou, Tao Jiang, et al. [10]	Retrospective Observational	Level 3
34952000	Pierina Navarria, Federico Pessina, et al. [39]	Prospective Phase II Clinical Trial	Level 2
35171292	Gaia Ninatti, Martina Sollini, et al. [40]	Systematic Review and Meta-Analysis	Level 1
35179134	Antonio Dono, Kristin Alfaro-Munoz, et al. [14]	Retrospective Observational	Level 3
35692047	A. Wick, A. Sander, et al. [5]	Retrospective Observational	Level 3
35716310	Jan-Michael Werner, Lena Wolf, et al. [42]	Prospective Randomized Controlled Trial (RCT)	Level 1
35948444	Lauren R. Schaff, Marina Kushnirsky, et al. [31]	Retrospective Observational	Level 3
35998208	Maximilian J. Mair, Annette Leibetseder, et al. [8]	Retrospective Case Series	Level 4
36215231	Michael M. Wollring, Jan-Michael Werner, et al. [23]	Retrospective Cohort Study	Level 3
36273731	Kepeng Liu, Xiaozu Liao, et al. [20]	Retrospective Observational	Level 3
36552087	Lea Scherschinski, Jubran H. Jubran, et al. [44]	SEER Database Analysis	Level 3
36598613	Tanja Eichkorn, Jonathan W. Lischalk, et al. [21]	Case Series	Level 4
36599113	Shawn L Hervey-Jumper, Yalan Zhang, et al. [7]	Retrospective Cohort Study	Level 3
36691100	Hongbo Liu, Lu Zhang, et al. [1]	Retrospective Cohort Study	Level 3
36852494	Andrew J. Scott, Luis O. Correa, et al. [37]	Retrospective Observational	Level 3
36964732	Sidonie Sauvageot, Julien Boetto, et al. [43]	Observational Study	Level 3
37147958	Dilek Erdem, Meral Gunaldi, et al. [38]	Retrospective Surgical Study	Level 3
37215951	Saksham Gupta, Noah L. Nawabi, et al. [12]	Experimental Study	Level 4
37216045	Luisa Allwohn, Josy Wolfgang, et al. [29]	Retrospective Cohort Study	Level 3
37370678	Julia Gilhodes, Adèle Meola, et al. [16]	Retrospective Cohort Study	Level 3
37435019	Jun Yin, Wenya Yin, et al. [24]	Retrospective Study	Level 3
37603235	Louise Carstam, Francesco Latini, et al. [32]	Retrospective Cohort Study	Level 3
37611077	Connor J. Kinslow, Ali I. Rae, et al. [11]	Retrospective Cohort Study	Level 3
37625215	Arshad A. Pandith, Wani Zahoor, et al. [13]	Retrospective Study	Level 3
37877960	Alexandra M Giantini-Larsen, Zaki Abou-Mrad, et al. [30]	Retrospective Observational	Level 3
37968476	Qinghui Zhu, Haihui Jiang, et al. [15]	Retrospective Observational	Level 3
38259678	Mashal Shah, Saad Bin Anis, et al. [41]	Retrospective Cohort Study	Level 3
38334907	Obada T. Alhalabi, Philip Dao Trong, et al. [35]	Retrospective Observational	Level 3
38579724	Avishay Spitzer, Simon Gritsch, et al. [22]	Experimental Study	Level 4
38845694	Colin M. Harari, Adam R. Burr, et al. [36]	Retrospective Cohort Study	Level 3
38971940	Yukyeng Byeon, Chaejin Lee, et al. [33]	Retrospective Cohort Study	Level 3
38981364	Zhi Xuan Ng, Eng Siew Koh, et al. [26]	Systematic Review and Meta-Analysis	Level 1
